# Pseudoaneurysm Development after Free Wall Rupture Post Myocardial Infarction

**DOI:** 10.3390/jcdd7030034

**Published:** 2020-09-07

**Authors:** Steven Douedi, Nasam Alfraji, Vandan D. Upadhyaya, Mihir Odak, Matthew Meleka, Muhammad R. Raza

**Affiliations:** 1 Department of Medicine, Jersey Shore University Medical Center, Hackensack Meridian Health, Neptune, NJ 07753, USA; Nasam.Alfraji@hackensackmeridian.org (N.A.); Vandan.Upadhyaya@hackensackmeridian.org (V.D.U.); Mihir.Odak@hackensackmeridian.org (M.O.); 2 Department of Cardiology, Jersey Shore University Medical Center, Hackensack Meridian Health, Neptune, NJ 07753, USA; Matthew.Meleka@hackensackmeridian.org (M.M.); Muhammad.Raza@hackensackmeridian.org (M.R.R.)

**Keywords:** pseudoaneurysm, wall rupture, echocardiogram, myocardial infarction, percutaneous coronary intervention

## Abstract

**Background:** According to the World Health Organization, cardiovascular disease is the number one cause of death globally, claiming millions of lives each year with an increasing prevalence. Myocardial infarction (MI) makes up a large sum of these deaths each year. While MI in itself is lethal, there are several complications that can increase the morbidity and mortality of an MI, such as left ventricular wall rupture and aneurysms. **Case Presentation:** We present a case of an elderly male with an extensive cardiac history who presented with a non-ST segment myocardial infarction (NSTEMI) managed with percutaneous coronary intervention. Hours after, he became hemodynamically unable and was found to have a pseudoaneurysm of the left ventricle. Despite aggressive efforts, his pseudoaneurysm ruptured and he ultimately succumbed to his condition. **Conclusions:** Left ventricular pseudoaneurysm is usually seen after myocardial infarctions with a rupture rate of up to 45% leading to a mortality rate of about 50%. While cardiac catheterization with left ventriculography is the gold standard for diagnosis, echocardiography can also be used as an alternative. Treatment is emergent cardiac surgery but still holds a high operative risk. Therefore, patients may be medically stabilized and managed prior to ultimate surgical intervention.

## 1. Introduction

Left ventricular wall rupture and aneurysm formation are uncommon, but critical structural complications more commonly associated post myocardial infarction [1]. With new advancements in technology and medical care, the incidence of both free wall rupture and aneurysm or pseudoaneurysm formation have been noted to be less than 1%. Non-ST-segment elevation myocardial infarctions (NSTEMI) are a small subgroup of these reported cases leading to structural complications, however, physicians must have a high index of suspicion for this challenging consequence to commence early intervention and reduce patient morbidity and mortality.

## 2. Case Presentation

**Figure 1:**
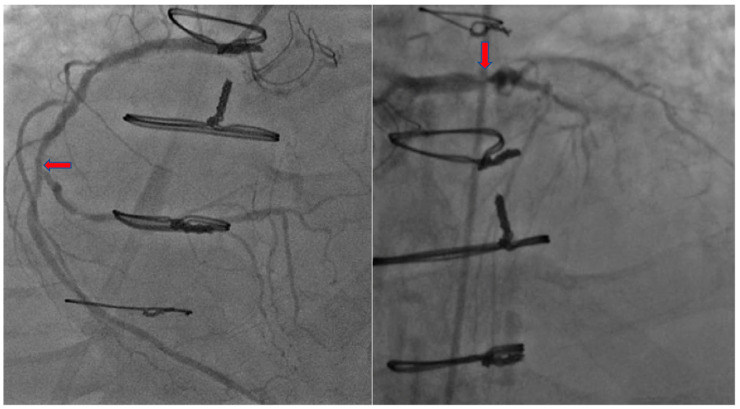
Coronary Angiography of right coronary artery (**left**) and left coronary artery (**right**).

**Figure 2:**
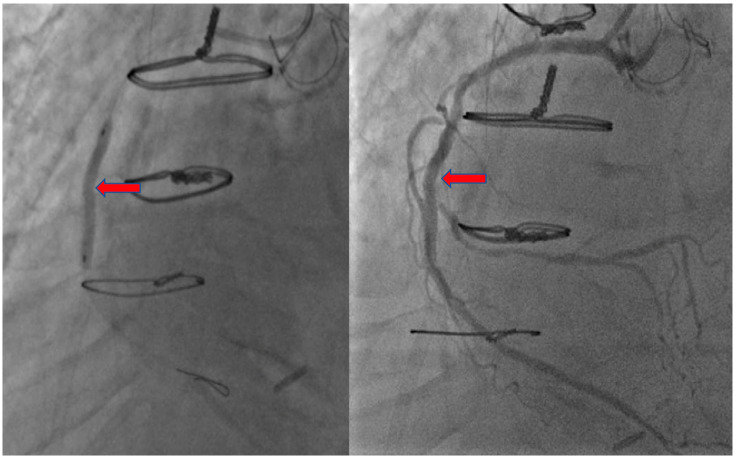
Coronary Angiography of right coronary artery with stent.

A 66-year-old male with an extensive cardiac risk factors and history, including two vessel coronary artery disease status post coronary artery bypass grafting 3 years prior to admission, aortic stenosis status post valve replacement, and peripheral vascular disease presented to the emergency department (ED), complaining of worsening shortness of breath over a 4 day period and bilateral leg swelling. He also states he had noted some substernal chest discomfort that has remained unchanging over about one week. In the ED, vitals were a temperature of 98.1 degrees Fahrenheit (36.7 degrees Celsius), blood pressure of 134/88 mm Hg, heart rate of 81 beats per minute, and oxygen saturation of 97% on room air. Laboratory testing was significant for a brain natriuretic peptide level of 1857 pg/nL and a troponin level of 1.87 ng/mL. Electrocardiogram (ECG) was obtained showing a normal sinus rhythm without ST-segment or T wave changes. He underwent an echocardiogram which revealed an ejection fraction (EF) of 30%, decreased from his echocardiogram one-year prior with an EF of 60%. He was started on intravenous (IV) diuretics for acute systolic congestive heart failure exacerbation. He was also started on IV heparin and loaded with 325 mg of aspirin then underwent successful percutaneous coronary intervention (PCI), where he was found to have severe native vessel and left main disease and received a drug-eluding stent to the mid right coronary artery, due to suspicion of being the culprit vessel (Figure 1 and Figure 2).

**Figure 3:**
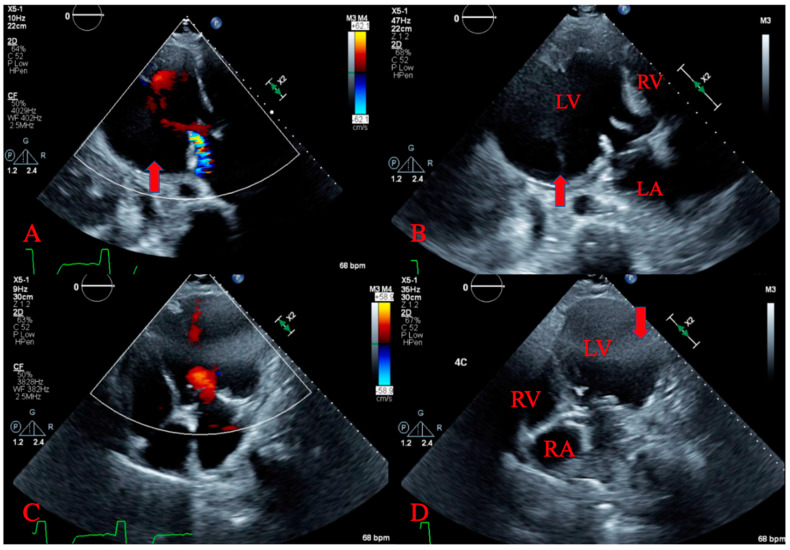
Transthoracic echocardiogram. (**A**,**B**) demonstrating left ventricular pseudoaneurysm with to and fro flow. (**C**,**D**) showing large loculated pericardial effusion.

Several hours later, he began to complain of shortness of breath, nausea, and the sensation of passing out. His vitals became unstable with a blood pressure of 82/48 mm Hg and heart rate of 48 beats per minute. He was intubated for airway protection and placed on vasopressors for hemodynamic stability and sent to the cardiac care unit. An ECG showed nonspecific intraventricular conduction delay and septal T wave abnormality possibly due to a septal ischemia. An emergent transthoracic echocardiogram was performed and revealed a large pericardial effusion overlying the left ventricle, and large pseudoaneurysm in the anterolateral wall with “to- and fro-” flow consistent with contained free wall rupture (Figure 3A,B). A large loculated pericardial effusion overlying the left ventricle representing free wall rupture was also seen (Figure 3C,D).

Cardiothoracic surgery was emergently consulted and, en route to the operating room, he again quickly decompensated and was deemed unstable and a poor candidate for surgery. After stabilization with vasopressors for hemodynamic support and mechanical ventilation, he was transferred to another facility 48 h later for emergent cardiothoracic intervention, and was considered for a heart transplant. Unfortunately, despite aggressive efforts, his pseudoaneurysm ruptured and he ultimately succumbed to his conditions and passed away.

## 3. Discussion

Atherosclerotic coronary artery disease is still considered the main cause of death worldwide [1]. Several complications are attributed to the increased morbidity and mortality in coronary artery diseases including arrhythmic, embolic/thrombotic, mechanical/structural, and ischemic involvement [1]. Although PCI, over the last 20 years, have significantly lowered mortality rates in acute myocardial infarctions (AMI), 30-day mortality still stands at 7.8%, due to the resulting complications [1].

Left ventricular free wall rupture and left ventricular aneurysm (LVA) formation are serious, yet rare, structural complications post myocardial infarction [1]. LVA can be classified as a true aneurysm versus pseudo-aneurysm (false aneurysm) [1]. The true LVA is defined as well-demarcated scarred wall containing necrotic myocardial muscle with akinetic or dyskinetic function in the setting of a healed transmural myocardial infarction [1]. While pseudoaneurysm is defined as ventricular free wall rupture that is contained by the overlying pericardial layer, and, unlike a true aneurysm, it does not contain endocardium or myocardium, which our case developed [1,2].

The structural complications can be explained by understanding the first two of four steps in wound healing after AMI [3]. The first step is the acute ischemic phase and occurs within 4–6 h after the ischemic where a transient increase in the compliance of the left ventricular (LV) wall is noted with a decrease in the contractility of the ischemic region [3]. This results in a progressive thinning of the scarred tissue and eventual stiffness of the infarcted region [3]. The second phase is the necrotic phase and lasts for 5–7 days after the event and is characterized by unchanged end-diastolic lengths of ischemic regions, indicative of even stiffer LV walls [3]. This is thought to occur due to degradation of titin proteins secondary to inflammatory response and edema surrounding the infarcted region [3]. The inflammatory degradation of structural proteins in the myocardium makes pseudoaneurysms and LV wall rupture a possible complication occurring during this phase of wound healing [3].

Prior to reperfusion therapy, heart rupture after AMI had an incidence of 6%, however, as of the most recent clinical trials and registries, the incidence has declined to 0.2–1% [4,5]. Free wall rupture does still carry a high mortality rate of up to 80%, as compared to other forms of heart rupture, such as septal rupture, which was 41% [5]. Cases of heart rupture, including free wall and septal, accounted for 5.6% of all hospital deaths and carried a 58% mortality rate, as compared to 4.5% in non-heart rupture cases [5]. In the Global Registry of Acute Coronary Events (GRACE) cohort trial—used to risk stratify patient’s mortality with non-ST-segment elevation myocardial infraction (NSTEMI), and which studied the incidence and factors associated with heart rupture in 60,198 acute coronary syndrome patients—the incidence of heart rupture was 0.9% in ST-segment elevation myocardial infarction (STEMI), while it was less common in unstable angina at 0.25%, and the rarest in NSTEMI at 0.17% [5]. In the same study, factors noted to be associated with higher risk of heart rupture are older age (mean age of 74 years old), female sex, previous stroke, ST-segment elevation, positive cardiac enzymes, higher GRACE score, higher heart rate and lower blood pressure at presentation [5].

Left ventricular pseudoaneurysm can be also be caused by trauma, cardiac surgery, and infections [2,6,7]. However, myocardial infarction (MI) is still considered the most common cause for LV pseudoaneurysm [7,8]. Clinical presentation may vary from anginal pain, symptomatic congestive heart failure, syncope, arrhythmia, cardiac rupture, tamponade and shock, to even asymptomatic in about 10–12% [2,6,7,8]. Diagnostic work up includes transthoracic/transesophageal echocardiography, magnetic resonance tomography, ventriculography, computed tomography, and radioisotope imaging [6,8]. While cardiac catheterization with left ventriculography is the gold standard for diagnosis, echocardiography is considered a great preliminary non-invasive diagnostic method for LV pseudoaneurysm [6,8,9].

Echocardiography can allow for visualization of myocardial discontinuity, contour and shape of saccular aneurysm pouches, and of echogenic thrombi located within the LV [10], suggestive of LV pseudoaneurysm. Echocardiography can also allow for visualization of the opening orifice of the aneurysm and for assessment of its size [10], and usage of color-flow doppler may reveal bidirectional flow through the orifice [11]. Pseudoaneurysms are also characterized has having narrow necks, with an end-systolic orifice diameter-to-maximum aneurysm diameter ratio of <0.5 [11]. An identification of these array of findings can provide a high index of suspicion for LVP via transthoracic echocardiography.

LV pseudoaneurysm cases were reported infrequently in the literature, and it is clinically uncommon due to high risk of rupture, up to 45%, and subsequent remarkable mortality [9]. On our literature review, the most reported presentations were congestive heart failure (36%), chest pain (30%), and dyspnea (25%); while sudden death was noted in 3% of cases [6,7,8,12,13,14]. Myocardial infarction was the most common underlying etiology for LV pseudoaneurysm. Inferior infarcts are considered a higher risk for LV pseudoaneurysm formation than anterior infarcts, and anterior pseudoaneurysms are noted to be less common than posterior pseudoaneurysms [6,7,8,12,13,14]. Regardless of the cause and presentation, once identified on echocardiography, goal directed care should be aimed at ensuring hemodynamic stability with intravenous fluids, inotropic agents, and small volume pericardiocentesis, as clinically indicated as a bridge to ultimate cardiac surgery [4,9].

## 4. Conclusions

Free wall rupture is a lethal complication seen uncommonly after a myocardial infarction. Presentations can vary from asymptomatic to severe cardiogenic shock with acute hemodynamic instability. In rare cases, free wall rupture can be contained forming a pseudoaneurysm, as seen in our patient, requiring a prompt use of diagnosis and management. Due to a significantly high risk of rupture, echocardiography is the most feasible non-invasive diagnostic method used for LV pseudoaneurysm, and surgery is considered the ultimate treatment in most reported cases.
